# Access to health care for children with neural tube defects: Experiences of mothers in Zambia

**DOI:** 10.4102/ajod.v5i1.267

**Published:** 2016-12-02

**Authors:** Micah M. Simpamba, Patricia M. Struthers, Margaret M. Mweshi

**Affiliations:** 1 Department of Physiotherapy, University of the Western Cape, South Africa; 2 School of Public Health, University of the Western Cape, South Africa; 3 Physiotherapy Department, University of Zambia, South Africa

## Abstract

**Introduction:**

In Zambia, all children born with neural tube defects requiring surgery need to be referred to a tertiary level hospital in Lusaka, the capital city, where the specialists are based. The aim of this study was to explore the experiences of mothers accessing health care who had recently given birth to a child with a neural tube defect.

**Methods and analysis:**

In-depth interviews were conducted with a purposively selected sample of 20 mothers at the tertiary level hospital. The interviews were audiotaped, transcribed verbatim and translated. Content analysis was used to identify codes, which were later collapsed into categories and themes.

**Findings:**

Five themes emerged: access to health care, access to transport, access to information, concerns about family and support needs.

**Discussion:**

Barriers to access to health care included geographical barriers and barriers linked to availability. Geographical barriers were related to distance between home and the health centre, and referral between health facilities. Barriers to availability included the lack of specialist health workers at various levels, and insufficient hospital vehicles to transport mothers and children to the tertiary level hospital. The main barrier to affordability was the cost of transport, which was alleviated by either family or government support. Acceptability of the health services was affected by a lack of information, incorrect advice, the attitude of health workers and the beliefs of the family.

**Conclusion:**

Access to health care by mothers of children with neural tube defects in Zambia is affected by geographical accessibility, availability, affordability and acceptability. The supply-side barriers and demand-side barriers require different interventions to address them. This suggests that health policy is needed which ensures access to surgery and follow-up care.

## Introduction

Global estimates of birth defects indicate that 7.9 million children are born with birth defects each year and of these 90% are born in low- and middle-income countries (Christianson, Howson & Modell 2006). Neural tube defects (NTDs) are the second most common group of serious birth defects, following cardiac abnormalities, which result in infant mortality and severe disability (Rofail *et al.* 2012). They are a group of congenital defects of the central nervous system, resulting from failure of the neural tube to close during the first few weeks of foetal development (Padmanabhan 2006). NTDs are classified according to the anatomical structures affected: the cranial structures, anencephaly (major part of the brain is absent) or encephalocele (protrusion out of the skull of sac-like meninges and brain tissue), or the spinal structures (spina bifida), meningocele (sac protrudes out of the spine) or myelomeningocele (sac contains spinal cord and nerves) (Bussuk & Kibar 2009). The defect is classified as closed if skin covers the defect, and it is classified as open if skin does not cover the defect. Children with NTDs, especially spina bifida, may survive with lifelong neuromuscular, orthopaedic and sometimes cognitive and language disabilities (Wallingford *et al.* 2013).

## Background

The prevalence of NTDs varies between and within countries and may depend on geographical location, genetics or race (Lumley *et al.* 2001; Mitchell 2005). The worldwide incidence of NTDs is estimated to range between 1.0 and 10.0 per 1000 births (Au, Ashley-Koch & Northrup 2010). A systematic literature review on NTDs (1990–2014) by Zaganjor *et al.* (2016) found that the reported incidence of NTDs varied greatly between and within regions. The regional incidence per 10 000 births was 11.7 in Africa, 21.9 in the Eastern Mediterranean, 9 in Europe, 11.5 in the Americas, 15.8 in South-East Asia and 6.9 in the Western Pacific. In hospital-based retrospective studies, an incidence of 7.5 per 1000 births was reported in Algeria (2004–2006) (Houcher *et al*. 2008), 3.5/1000 births in Sudan (2003–2004) (Elsheikh & Ibrahim 2009) and 2.2/1000 births in Nigeria (2011–2013) (Nnadi & Singh 2016). A retrospective study at a paediatric neurosurgical centre in Kenya (2005–2010) reported the incidence of spina bifida and encephalocele as 3.3/10 000 live births (Githuku *et al.* 2014).

There is a paucity of data on the prevalence of NTDs in most African countries, with information only from hospital-based studies, thus not reflecting the actual prevalence (Rabiu & Adeleye 2013). In most high-income countries, there has been a decline in the prevalence of NTDs arising from advancement in prenatal diagnosis, serum alpha-fetoprotein tests, termination of affected pregnancies and folic acid supplementation amongst women of childbearing age (Fletcher & Brei 2010). Despite overwhelming evidence from high-income countries on prevention of NTDs, most countries in sub-Saharan Africa do not have policies on the prevention of NTDs (Adeleye, Dairo & Olowookere 2010; de Paul Djientcheu *et al.* 2008; Lumley *et al.* 2001). South Africa is the only country in Africa which has reported the implementation of food fortification and the subsequent reduction in the prevalence of NTDs after fortification of maize meal, the staple food (Sayed *et al.* 2008).

Besides making advances in the prevention of NTDs, high-income countries have also made advances in the management of NTDs and their complications, leading to more children surviving into adulthood (Bowman & McLone 2010). In high-income countries, in utero surgical repair for the unborn child with myelomeningocele reduces the incidence of hydrocephalus, and results in significant improvements in neurological function (Copp, Stanier & Greene 2013). When a child is born with an open NTD, surgical closure is recommended within the first 24 h (Lazareff 2011).

However, in low- and middle-income countries, specialised surgery is only available in the biggest cities. This is a challenge for parents of children with NTDs who are usually from lower socio-economic background and living in rural areas (Adeleye *et al*. 2010; Farmer & Kim 2008). Late presentation for surgery leads to high mortality rates and severe impairments for those who survive (Lazareff 2011; Miles 2006).

There are many barriers to surgical care in low- and middle-income countries. A systematic review by Grimes *et al.* (2011) identified the following barriers: the distance to hospital, poor roads, a lack of suitable transport, the lack of local resources and expertise at health facilities; direct and indirect costs related to surgical care; and fear of undergoing surgery and anaesthesia. A study in Nepal reported the limited availability of services, financial difficulties, and fear or distrust of service providers as barriers to surgical care (Nagarajan *et al.* 2015). In Cameroon, de Paul Djientcheu *et al.* (2008) found poverty and cultural beliefs of family members to be barriers to accessing health care.

The barriers faced by people in low- and middle-income countries in gaining access to health care can be explained using the four dimensions of the Access to Care Framework, namely, availability, accessibility, affordability and acceptability (Jacobs *et al.* 2012; Penchansky & Thomas 1981; Peters *et al.* 2008; Ricketts & Goldsmith 2005). The definition of ‘access’ used in this study is the one adopted by Jacobs *et al.* (2012 289), from Peters *et al.* (2008), which describes access to health services as ‘the timely use of service according to need’. These authors describe the four dimensions of access to care as availability (the existence of health care personnel and resources), accessibility (the geographical relationship between the health facility and the location of the user), affordability (the costs involved) and acceptability (attitudes of users towards providers and vice versa).

### Statement of the problem

In Zambia, all children born with NTDs and in need of surgery have to be transported to Lusaka for surgical management because specialist surgery is only available at the University Teaching Hospital (UTH) and Beit Cure Hospital, both of which are in Lusaka, the capital city of Zambia. There has been no study carried out in Zambia to explore the experiences of mothers of children with NTDs in relation to accessing health care services.

### Aim

The aim of this study was to explore the challenges faced by mothers of children with NTDs in accessing specialised health care services at UTH.

## Methodology

### Study site

UTH is the biggest hospital in Zambia, situated approximately 4 km east of the centre of Lusaka. This hospital receives referrals from all the nine provinces in the country and, as the country’s specialist centre, also acts as the principal training institution for all health and allied disciplines. This study was conducted in the paediatric surgical ward at UTH, namely, Ward D01.

### Population and sampling

The study population consisted of mothers of children with NTDs who were admitted to ward D01 between September and December 2011 (the period of data collection). The study sample included 20 mothers who were selected using purposive sampling so that mothers from all of the nine provinces could be represented. Sample size was based on data saturation, which was considered reached if interviews with two mothers from the same province yielded similar codes during analysis. However, if different codes emerged from the data, a third mother from that province was interviewed. Using these criteria, two mothers were interviewed from each of five provinces, three mothers from each of three provinces and one mother from one of the provinces, constituting a total of 20 mothers.

### Data collection

In-depth, face-to-face interviews were conducted in a private room in the ward, using a semi-structured interview guide (Ryan, Coughlan & Cronin 2009). The interview guide had two broad open-ended questions. The first question was asked to the mother to narrate, in the form of a story, what she experienced from the birth of the baby through to the baby’s admission to UTH. The second question was related to access to services following discharge. The interviews were conducted in four local Zambian languages, namely, Chi Bemba, Chi Tonga, Chi Nyanja and Si Lozi, and one interview was conducted in English. Over the period of data collection, new admissions of children with NTDs were identified through the ward’s admission register.

### Data analysis

The audiotaped data from the interviews were transcribed verbatim. The interviews conducted in local Zambian languages were transcribed and translated into English by a professional translator. The analysis approach was thematic analysis as described by Green and Thorogood (2009). Using a deductive approach, codes were identified, which were subsequently grouped into categories (Graneheim & Lundman 2004) from which themes were generated (Bradley, Curry & Devers 2007; Graneheim & Lundman 2004).

### Trustworthiness

Member checking was undertaken to strengthen credibility, by returning to the mothers whose children were still in the ward after the initial analysis of their interviews. They could verify whether the interpretations, and subsequent codes and themes, accurately represented their views (Green & Thorogood 2009). Prolonged engagement with participants was possible because the mothers were present in the ward for lengthy periods. The detailed descriptions of the context, participant characteristics, data collection methods and process of analysis are provided to support transferability, facilitating the repetition of the study in similar context (Bhattacherjee 2012; Morrow 2005; Zhang & Wildemuth 2009).

## Ethical considerations

Ethical clearance was obtained through written permission from the Senate Research Ethics Committee of the University of the Western Cape (Research project no: 11/5/11) and the University of Zambia Research Ethics Committee (Research permission no: 013-07-11). Written permission to conduct the study at UTH was granted by the hospital management and the nursing officer in charge of ward D01. The purpose of the study was explained to the mothers, using an information sheet translated into their home languages, and informed written consent was obtained from those who agreed to participate and have the interview audiotaped. The mothers were assured of confidentiality and that the information from audiotapes would only be used for this study, and that audiotapes would be destroyed after finalisation of the study.

## Findings

### Demographic characteristics

A total of 20 mothers participated in this study. They were aged 19–36 years (mean = 26 years). Of the mothers, 15 were from rural areas and dependent on subsistence farming. Four of the mothers were self-employed, selling vegetables at the market, making tablecloths, and one making a ‘home-brew’. Fifteen mothers were married; 11 husbands were subsistence farmers; four others were employed: a taxi driver, a gardener, a plumber and one self-employed person making basins. The five other mothers included in the study were divorced or widowed women and one was a 19-year-old school student. All mothers and most husbands would have had a very low income.

The children were aged between 1 day old and 9 months on date of admission. There was a predominance of male (*n* = 14) children in comparison to female (*n* = 6) children. The children’s NTDs included myelomeningocele (*n* = 10), meningocele (*n* = 6) and encephalocele (*n* = 4).

### Emerging themes

Five themes emerged from the analysis related to the experiences of the mothers in accessing health care:

- access to health facilities
- access to transport
- access to information
- concerns about family
- support needs.

### Access to health facilities

Mothers indicated that their experiences of accessing health facilities were a challenge, especially factors related to the birth of the child and the referral system.

Seven mothers had given birth at home (six from rural areas and one from Lusaka) and 11 mothers in a health facility (two in a clinic, eight at a primary level hospital and one at a secondary level hospital). Two mothers had given birth on the way to the health facility: one in a stranger’s home in the urban area and the other in the bush in the rural area.

‘We were walking from home on our way to the hospital, then I felt that I could not manage to reach the hospital … We asked for assistance from one of the houses on the way and I delivered there.’ (Mother 28 years, child with lumbar meningocele)

‘As we were walking, I just felt my legs were heavy and I could not walk so my sister in-law just put the chitenge [*cloth wrap women wear*] on the ground and I delivered there … Yes, in the bush … home was very far away and the clinic was also far away … After the baby was born, we continued walking to the clinic.’ (Mother 23 years, child with occipital encephalocele)

All the children needed to be referred to the next level of health facility until they reached the tertiary level hospital, as hospital staff at lower levels did not have the skills to work with children with NTDs. These facilities included the local health clinic (the health centre in an urban area or the health post in a rural area), the primary or first level hospital, the secondary level hospital and the tertiary level hospital (UTH) in Lusaka.

‘So they [*nurses*] said, this problem, we can’t do anything here, so you will go with other patients to Ndola [*secondary level hospital*] using the hospital vehicle.’ (Mother 28 years, child with lumbar meningocele)

‘The person at the clinic sent a radio message to [*name of hospital*] and the ambulance came to pick us. I asked if I could go home to pick up clothes for the baby but they said no.’ (Mother 23 years, child with occipital encephalocele)

Although all the children were immediately referred from the local health clinic to a primary level hospital, eight mothers reported that doctors at this hospital subsequently sent them home. Some needed to go home to find transport money:

‘I was admitted and after two days, I was given a letter to take to Lusaka … So we had to go back [*to the village*] to look for transport money to go to Lusaka.’ (Mother 21 years, child with encephalocele)

One mother was told she must go home, as her child was too small to be transported to Lusaka. Some children were sent home despite having unstable lesions:

‘So we went home and we stayed there. Then we noticed that the head of the baby was getting bigger. So we went back to the hospital … when I went back, they told me “we advised you to go to Lusaka”.’ (Mother 25 years, child with lumbar meningocele)

### Access to transport

Transport was one of the greatest challenges that most of the mothers experienced. They talked about difficulties with finding transport from home to the local health facility. While some mothers used public transport, others walked or were given a lift on a bicycle:

‘I was carried on a bicycle from home to the hospital.’ (Mother 20 years, child with large encephalocele died a few days after surgery)

It was costly to find transport from the local clinic to the primary level hospital to the secondary level hospital and, subsequently, to the tertiary level hospital (UTH). Although some mothers were given free transport by the health facility, one mother paid for the fuel for the hospital vehicle:

‘The hospital staff said that they didn’t have money for fuel … They told us to put fuel in … the hospital vehicle so that we could travel in it to Lusaka. So we gave them some money.’ (Mother 19 years, child with lumbosacral myelomeningocele and hydrocephalus)

The staff at two facilities contributed their own money to enable two mothers travel to Lusaka with the children:

‘The doctor said that where you are going, you will need some money. So the staff at the hospital contributed some money.’ (Mother 36 years, child with lumbar myelomeningocele)

At other health facilities, mothers were not given any help with transport and were told to find their own transport to the referral hospital. One of these mothers went to the local authority in the town, where a vehicle was provided to transport the baby and her husband with her to Lusaka.

‘After three days, the [*baby’s*] condition was just getting worse. I said this head is becoming worse, so I went back … So I left home and I said to myself “Let me just go to the DC *[District Commissioner]*”.’ (Mother 25 years, child with lumbar meningocele and hydrocephalus)

One mother went home and stayed there until some strangers, visiting the village, gave her some money for transport:

‘We did not have money to go to Lusaka so we went back home and stayed for three months. Later some white people came to our area and they gave us some money to take the child to Lusaka.’ (Mother 21 years, child with occipital encephalocele)

One mother had to sell the family’s cash crop of maize to obtain the transport money. Another mother had travelled by bicycle to the neighbouring country, hoping that she could have easier access to appropriate health care.

‘Someone advised us to go to Malawi … and there they told us to go to Lilongwe [*the* *capital* *city*] … We had to come back because we didn’t have money to go there.’ (Mother 25 years, child with lumbar meningocele)

Almost all mothers were concerned about how they would afford transport to go home when the child was discharged from UTH. Even the mothers who had been assisted with getting transport to Lusaka were worried about how they would go home, not knowing whether the referring hospital would send an ambulance to take them home or whether those who had assisted them (for example, the mother who was assisted by the District Commissioner) would send transport to fetch them:

‘So they said you should call but each time we call they are outside coverage area [*no telephone reception*] … So now we are worried because we don’t know where we will find money to go back when the baby gets better.’ (Mother 24 years, child with myelomeningocele and hydrocephalus)

When asked about accessibility of health care services near their homes, mothers who lived near the first or second level hospitals indicated that they would not have any problems with access, but mothers who lived far from these hospitals expressed concerns about transport to these facilities. The majority of mothers indicated that they would experience transport difficulties if they were expected to return to Lusaka for a follow-up appointment.

‘… but if they operate and say we should come back to Lusaka for review, then there will be a problem with transport.’ (Mother 22 years, child with myelomeningocele)

‘I cannot afford to move from Mansa (town in Luapula Province) to this place. It is just too much [*she laughs*].’ (Mother 24 years, child with nasal encephalocele)

### Access to information

Most of the mothers expressed concern about the lack of information and uncertainty they were experiencing concerning their child’s lesion. Despite attending antenatal care, the mothers were not aware that they were going to give birth to a child with an NTD; therefore, learning about their child’s neurological condition was a shock.

‘The time I was pregnant they thought I had twins, but when I delivered that is when they discovered the problem … I delivered well without any problems but when the child came out, I noticed this thing on the head.’ (Mother 20 years, child with large occipital encephalocele who died few days after surgery)

‘I used to go for antenatal checks but they never mentioned about it … So I had little knowledge about this and it is very difficult, very distressing when you are going through this.’ (Mother 28 years, child with lumbosacral myelomeningocele)

Mothers were concerned that the health workers were not explaining things to them. The mothers said they had no prior knowledge about NTDs and wanted to know the cause. While some mothers wanted to know what kind of surgery their children would have, those whose children had not had surgery could not understand why children with similar conditions were already having surgery:

‘I have noticed that all of my friends’ babies with this problem are being operated on and I thought that even my baby will be okay if they operate.’ (Mother 34 years old, child with myelomeningocele)

Some mothers also talked about the need to improve their own skills, and for more information on how to take care of the children. Most mothers were concerned about the future of their children, as they wanted to know whether the child would be able to sit or walk:

‘I just want to find out if the baby will be able to sit because the wound is somewhere here where he’s supposed to sit, so I’m wondering how he can sit.’ (Mother 25 years, child with lumbar meningocele)

### Concerns about family

Mothers expressed concerns related to their families. Unaware of the unborn baby’s neural lesion and that they would need to go to the tertiary level hospital in Lusaka immediately after the birth, mothers went to the usual health facility to give birth. They had made no provision for someone to care for their other children for a lengthy period. While some mothers had left their other children in the care of relatives, some were worried, as they had not had the opportunity to make such arrangements:

‘I just left the other children with no one to take care of them.’ (Mother of two other children, 23 years old, whose child with occipital encephalocele was born in the bush on the way to hospital)

The tertiary level hospital allowed only one person to spend the night next to the child’s bed: in general, it was the mother who was breastfeeding. When a husband had accompanied the mother to Lusaka, he would have to find his own place to sleep and food to eat. This increased the mother’s anxiety.

‘My husband is suffering a lot and sometimes he sleeps outside and sometimes they chase him. He has nowhere to stay … Yesterday I don’t know where he slept. The watchman took him … Yes, they just took him outside, so he went and slept there.’ (Mother 25 years, child with lumbar meningocele and hydrocephalus)

‘He just comes to see me, then he goes to the inter-city bus station to sleep, then he comes back in the morning.’ (Mother 20 years, child with large occipital encephalocele who died few days after surgery)

Some mothers were concerned about their families’ beliefs about the child having an NTD. One 25 year-old mother from the Eastern Province gave birth at home to a child with a lumbar meningocele (not an open lesion). Her husband and her in-laws refused to let her go to the hospital, instead applying traditional herbs to the lump on the child’s back. It was only after a week, having convinced the family, that she could take the baby to the clinic where she was referred to the hospital. The nature of the family’s beliefs and the herbs used were not investigated.

### Support needs

Mothers described the need for both family support and government support.

A number of mothers received support from their families prior to admission at UTH. When financially possible, the extended family assisted mothers who needed to pay for transport to Lusaka.

‘So my relatives said that since this baby is supposed to go to Lusaka, we are going back to the village to look for money so that we can travel well to Lusaka.’ (Mother 19 years, child with lumbosacral myelomeningocele)

Most mothers from rural areas had a family member, frequently the husband, who accompanied them to the primary and secondary level facilities and then to Lusaka, to give them support. However, some mothers whose relatives stayed a long way from Lusaka said they received no family support. During their stay at UTH, some mothers received support from relatives in Lusaka, including being visited by them, given food and help with taking care of the baby.

‘My sister in-law is helping me. At night I go home [*to her relative’s house*] and wash the wound [*had C-section*], then I come back early in the morning.’ (Mother 28 years, child with lumbosacral myelomeningocele)

Despite having relatives in Lusaka, some mothers did not have any support from them as these relatives were unwell or the family relationship was difficult.

‘You know I was so heart-broken because this is the person I thought was going to keep me … So I can’t stay with her.’ (Mother 36 years, child with lumbar myelomeningocele)

Additionally, family members played an important role in taking care of the mothers’ other children who had been left at home.

Some health facilities had supported the mothers by organizing transport for them. At the UTH, both the mothers and those who had escorted them were provided with lunch and supper.

When asked how the government should help other mothers who are in a similar situation, the mothers said the government needs to ensure that children with NTDs are transported to the referral hospitals as soon after birth as possible. Hospitals needed to provide transport, or the social welfare should provide transport money, to those who could not afford it.

‘But there must be social welfare to assist with transport money so that the baby is quickly taken to the hospital … but you know in our areas, such services, they say they don’t have enough money.’ (Mother 36 years, child with lumbar myelomeningocele)

Furthermore, one mother wanted the government to provide wheelchairs for children who were unable to walk.

## Discussion

The demographic characteristics of mothers in the current study are similar to those in other studies in Africa. The mean age of mothers in the current study was 26 years, and most of them were from poor socio-economic communities dependent on subsistence farming. A record review at the two hospitals in Zambia providing surgery for children with NTDs indicated that the most common age group was 1–6 months old, with 61% of children having myelomeningocele (Mweshi *et al.* 2011). A study in Kenya reported a mean age of 8.5 months at the time of the initial operation (Margaron *et al.* 2010). Similar findings were reported in Nigeria by Adeleye *et al.* (2010), who found that the mean age of mothers of children with NTDs was 28 years and that most of them were from poor socio-economic backgrounds.

The findings of this study are discussed using the four dimensions of the Access to Care Framework, accessibility, availability, affordability and acceptability, as described by Jacobs *et al.* (2012) and Peters *et al.* (2008) and the supply- and demand-side barriers affecting access, as described by Jacobs *et al.* (2012).

### Accessibility

In rural areas of Zambia, the geographical distance is a barrier for poor people to access health care, including at local health centres. As described by Jacobs *et al.* (2008), this is a supply-side barrier to access, as the locations of services are too far from the population served. In Zambia, it is estimated that about 99% of people in urban areas live within 5 km of a health facility, while in rural areas only 50% are within this range (MOH [Zambia] n.d.). In this study, mothers were not aware that their child had an NDT; therefore, this was not a factor in deciding where to deliver. Eight of the nine mothers who did not deliver at a health facility lived in the rural areas. Seven mothers delivered at home and two mothers delivered on their way to the health facility. Three of the mothers who did not deliver at a health facility gave birth to children with myelomeningocele, predisposing their babies to infection. According to Shehu and Ameh (2004), in sub-Saharan African countries, most babies with NTDs are delivered at home with risk of infection as a result of rupture of the myelomeningocele. Those with hydrocephalus may experience asphyxia arising from obstructed labour and risk of brain damage.

According to the Zambia Demographic and Health Survey (CSO, MOH & ICF International 2014), an estimated 67% of all deliveries in Zambia are at a health facility. However, when comparing urban and rural areas, it is found that 89% of urban births in Zambia take place in a health facility, while only 56% of rural births occur at a health facility. Houweling *et al.* (2007) add that home deliveries are common in low- and middle-income countries, especially amongst women from poor socio-economic backgrounds. According to de Groot (2008), the main reasons for home deliveries are the distance required to walk or the time required to travel to the facility.

Geographical distance from home to health facility has been identified as a major barrier to health care in low- and middle-income countries (Al-Taiar *et al.* 2010). A study in South Africa by Harris *et al.* (2011) reported that this was a greater barrier in rural areas and in poorly resourced provinces. Similarly, a systematic review of studies conducted in low- and middle-income countries (1990–2006) identified the place of residence, distance and transport to the health facilities as the main factors affecting the use of antenatal care services in low- and middle-income countries (Simkhada *et al.* 2008). A systematic review of socio-economic differences in morbidity and access to health in Uganda found that many studies identified distance from a health facility as one of the most common barriers to access (Kiwanuka *et al.* 2008).

Although distance to the health facility has been found to be a major reason for home delivery, according to a study in Zambia by Sialubanje *et al.* (2015), there are other reasons which include a lack of money for transport and the requirement to bring baby clothes and food while in hospital. Similarly, a study in Tanzania reported that the most common reasons given for home deliveries were the distance to the health facility and a lack of money, including a fear of caesarean section and a lack of privacy in the labour room (Mrisho *et al.* 2009).

Accessibility is also determined by transport, which is essential when a pregnant woman is unable to walk to the health facility to give birth. Jacobs *et al.* (2012) described this difficulty with transport, including both the distance between the home and the health facility and the public transport available, as a demand-side barrier to access. Unlike all the mothers from rural areas, the three mothers in this study who were living in Lusaka where surgery could take place reported that their child was referred to UTH within 24 h of birth.

Follow-up visits after surgery are important for children with NTDs, especially those with myelomeningocele and hydrocephalus, because it increases their chances of survival (Warf 2011). In Uganda, where children with myelomeningocele were followed up postoperatively by a community-based support person, the children had a higher 5-year survival compared to those who were not observed by the programme (Warf, Wright & Kulkarni 2011). In this study, most mothers from rural areas said that it would be difficult to return to Lusaka for a follow-up appointment because of the distance to the hospital and the cost of transport. These reasons are consistent with what has been found in other studies in low- and middle-income countries where follow-up visits are deterred by distance and transport costs (de Paul Djientcheu *et al*. 2008; Pirani *et al.* 2009).

### Availability

The inequitable distribution of health workers and physical infrastructure and the consequent lack of capacity to provide quality health care have been reported on by the Zambian government (MOH [Zambia] n.d.). At each level, access to health care is affected by the availability of skilled health workers, essential drugs and medical equipment. None of the mothers in this study had a prenatal diagnosis of NTD. According to reports from other African countries, even where prenatal testing is performed, it frequently fails to identify congenital anomalies (Adeleye *et al.* 2010; de Paul Djientcheu *et al.* 2008; Rabiu & Adeleye 2013). A prospective study of central nervous system anomalies in Nigeria reported that although 80% of the mothers had prenatal ultrasound, the anomaly was only diagnosed in 14% (Adeleye *et al.* 2010). A similar study in Nigeria reported that although 97% of the mothers of children with central nervous system anomalies had prenatal ultrasound, the detection rate was only 24.5% (Idowu & Olawehinmi 2012). The shortage of skilled health workers, who are able to make a correct prenatal diagnosis, is a supply-side barrier to access to health care (Jacobs *et al.* 2012).

In general, after referral, hospital transport was not available for the mothers at the primary level health facility, but was available at the secondary level facility. This was a supply-side barrier. The mothers wanted all the health facilities (as government institutions) to provide this transport on referral. Having hospital transport available is an important factor in accessing health care, particularly in rural areas. Furthermore, the mothers, including those who had been transported in a hospital vehicle to the tertiary level hospital, were very worried that there would be no hospital transport provided for them to return to the rural areas.

### Affordability

Peters *et al.* (2008) describe affordability, which is directly associated with poverty, as one of the most important determinants of access. Affordability relates to financial barriers to access, including both direct and indirect costs. A systematic review on barriers to surgical care in low- and middle-income countries identified the direct costs as those that are related to care: transport, hospital stay, surgery, drugs, laboratory tests and medical supplies. Indirect costs included the loss in productivity and income, and the cost of a caregiver (Grimes *et al.* 2011). Although health care services for children are free in Zambia, the direct and indirect costs can be demanding, especially for those from rural areas. According to the World Bank (2016), Zambia, a lower middle-income country, has a very unequal income distribution, with 60% of the population living below the poverty line and 42% considered to be in extreme poverty. The Zambia National Health Strategic Plan (2011-2015) states that poor people from rural areas face access barriers, including transport costs, time costs, and food and accommodation for in-patients and relatives (MOH [Zambia] n.d.).

Transport costs are a major barrier to access health care services amongst the poor and those in rural areas (Goudge *et al.* 2009; Harris *et al.* 2011). In this study, the major direct cost was transport, including the initial transport to the local health clinics and subsequent transport costs when the child was referred to the secondary and tertiary level hospitals. As described by Peters *et al.* (2008), this cost was related to the distance to the health facilities, in particular affecting mothers from rural areas and those who were not assisted with hospital transport.

Most of the mothers in this study had no source of cash income and relied on subsistence farming. One mother sold the maize, which the family expected to use as food in the coming months, to pay for her transport to Lusaka. Selling household assets or borrowing to pay for health care services is common in low- and middle-income countries where people, mostly the poor, use out of pocket money to finance health care services (Alam & Mahal 2014).

When mothers were accompanied by their husbands, there was no one to work in the fields, thus affecting income and food for their families, which is an indirect cost. Health service utilisation is the lowest amongst poor populations (O’Donnell 2007; Peters *et al.* 2008). A systematic review of socio-economic differences in morbidity and access to health in Uganda found that many studies identified distance from a health facility as one of the most common barriers to access (Kiwanuka *et al.* 2008). The cost of transport for the mothers is a demand-side factor related to affordability that is directly associated with distance from a health facility (Jacobs *et al.* 2012). Because of private transport costs, mothers rarely had any expectation of being able to return to UTH for follow-up visits.

Mothers who received transport support from the referring hospitals were able to reach Lusaka in good time despite coming from poor socio-economic backgrounds. Similarly, Penny *et al.* (2007) reported that in Uganda many children with motor impairments from poor rural populations were able to access the health services when they received financial and transport support. As Thiede and McIntyre (2008) noted, health care financing can have a significant effect on affordability by giving poor communities the opportunity to access health care without incurring any costs.

### Acceptability

The cultural practices in a particular society can be demand-side barriers affecting access to health care. Women’s lack of decision-making power has been found to delay access to health services, and may contribute to the high mortality rate amongst children in poor areas (Bronsard *et al.* 2008; Fantahun *et al.* 2007). In a study in rural Zambia, Sialubanje *et al.* (2015) found that women’s lack of decision-making autonomy and their dependence on their husband and other family members for the final decision were reasons for home deliveries. Home births are common in Zambia despite attempts to phase out traditional birth attendants. Regardless of the mother’s choice, many traditional families insist that a traditional birth attendant delivers the baby at home. In a study on access to and utilisation of health services for the poor in Uganda, Kiwanuka *et al.* (2008) reported that most poor women used traditional and untrained health personnel to assist their delivery. In this study, in addition to the home births, one mother reported that her husband’s family delayed her taking the child to the health facility as they wanted to apply herbs directly onto the lesion on the child’s back.

Most studies exploring the needs of parents of children with disabilities have reported access to information as one of the most important aspects mentioned by parents (Palisano *et al.* 2010; Resch *et al.* 2010). In this study, after delivery mothers did not have any understanding of what was wrong with their child. In a systematic review to understand the burden of spina bifida on caregivers, it was reported that at the time of the initial diagnosis, about 53.3% of mothers of children with spina bifida did not know that, in the future, their children would have bowel problems (Rofail *et al.* 2012). According to Resch *et al.* (2010), providing information to parents on the type of disability and services available at the time of diagnosis helps reduce parental stress. In a study conducted in South Africa, caregivers of children with learning disabilities reported that their lack of knowledge about their child’s condition was one of the main causes of their experiences of distress and anxiety when caring for their children (Sandy, Kgole & Mavundla 2013). Giving information during the initial diagnosis helps families to plan for their child’s future, learn of the services available and understand the long-term implications of their child’s condition (Jeglinsky, Autti-Rämö & Carlberg 2012; Palisano *et al.* 2010). Provision of information is a supply-side factor that can be used to facilitate acceptability of health care services amongst mothers of children with disabilities, and to increase utilisation of health care services for their children. Limited education may be a demand-side factor affecting access when mothers do not recognise illness or the potential benefits of getting health care (O’Donnell 2007).

The mothers’ social responsibilities, or the cultural and social distance between health care services and users, as described by Simon (2008), are another factor that can affect acceptability of the service. In this study, most of the mothers were concerned about the children who remained at home, while those women who were escorted by spouses were worried about the living conditions of their spouses in terms of accommodation and food. This is in line with the findings of Peters *et al.* (2008), who noted that, apart from worrying about the condition of the child, parents of hospitalised children in low- and middle-income countries also worry about other things such as cost of food and accommodation during the child’s hospitalisation. Furthermore, a systematic review on barriers to surgical care in low- and middle-income countries reported that the absence of a person to escort the family member to the hospital was a stronger factor in preventing access to surgical care than fear of surgery (Grimes *et al.* 2011).

In low- and middle-income countries where some hospitals do not provide meals or accommodation for patients and caregivers, family support is an important factor in terms of acceptability (Gyasi, Amoaku & Asamany 2007). Although the hospital provided meals for the mother and the person who escorted her, some mothers in this study who were accompanied by their husbands to Lusaka experienced an additional concern about their spouse’s living conditions. Thus, the absence of relatives near the urban referral hospitals may be considered a demand-side factor that affects access to health services by reducing compliance to referral.

At times the attitude of the health care workers, which is a supply-side factor, may have affected referral in this study. While some health workers recognised children born with NTDs require immediate referral to specialised services, at other hospitals health workers sent the children home. Whether or not this incorrect advice resulted from inadequate knowledge or negative attitude, a delay in receiving specialised treatment may lead to a deterioration of the condition, and increased operative and mortality risks (Komolafe, Komolafe & Adeolu 2008). Despite this, there were occasions where the attitude of the health personnel was a facilitator, for example, when they contributed money to pay for the mother and child’s transport to the tertiary level facility for surgery.

Although every attempt was made to ensure trustworthiness of the data, there were some limitations including the language barrier between the researcher and four of the participants whose languages the researcher was unable to speak. Research assistants, who were able to speak the mother’s language, conducted the interviews. However, this would have affected the depth of the data collected as well as the interpretation of nuances within the data.

The second limitation was the study sample, which only included mothers who had managed to report to the tertiary level hospital. This introduced a sample bias into the findings, as there may be mothers who found difficulties with access to the tertiary facility were too great. A study that includes mothers at primary and secondary level facilities might provide additional information on their experiences.

## Conclusion

Through the use of qualitative methodology, this study has given voice to mothers of children with NTDs in Zambia by describing their experiences of getting access to health care and the tertiary level health services for their children. Ensor and Cooper (2004) have argued that the demand-side barriers may be a greater factor in reducing access to health care in poor and vulnerable populations than the supply-side barriers; however, effective methods for reducing them have not been evident. Important demand-side barriers for rural mothers included geographical inaccessibility or distance between home and the health facility and limited public transport to health facilities. Other demand-side barriers that affected the acceptability of the service included the inability of mothers, who were subsistence farmers, to afford public transport, and the attitudes and cultural practices of the mother and father’s families concerning the health services and an absence of supportive relatives near the health facility. Supply-side barriers included the distance between health clinics and homes in rural areas, the absence of health workers with appropriate knowledge, skills and a caring attitude at each health facility level, information not being provided to mothers and the lack of hospital transport affecting access to the next facility level, following referral.

Interventions to improve access to health facilities in Zambia need to differentiate between supply-side and demand-side factors in order to ensure that newborn babies with NTDs have access to quality health care. A holistic approach is needed. The mothers recommended that the government should make transport a priority. While some factors can be addressed by the Zambian Ministry of Health, other government departments such as the transport department need to become involved. Furthermore, the training institutions need to take note of the findings and use them to inform their curricula. The development of such policies in other African countries with similar gross domestic products might facilitate access to health care for children born with NDTs.

Access to tertiary level facilities for surgical intervention is essential for babies born with NTDs. If closure of an open lesion is to be performed within 24 h, as advised by Lazareff (2011), there is an urgent need for an effective referral system and the provision of transport. Furthermore, as Warf (2011) has argued, following studies in Uganda, not only is surgery important, but also follow-up through programmes such as community-based rehabilitation have been found to significantly increase the chances of survival. As McQueen *et al.* (2010) state, essential surgical care needs to be considered as a basic human right. This study helps to emphasise that governments in sub-Saharan Africa, including Zambia, must develop health and other policies to ensure equal access to health care, including surgery and follow-up care.
